# Evaluating HIV Prevention Programs: Herpes Simplex Virus Type 2 Antibodies as Biomarker for Sexual Risk Behavior in Young Adults in Resource-Poor Countries

**DOI:** 10.1371/journal.pone.0128370

**Published:** 2015-05-26

**Authors:** Juliane Behling, Adrienne K. Chan, Clement Zeh, Carolyne Nekesa, Lucie Heinzerling

**Affiliations:** 1 Friedrich-Alexander-University Erlangen-Nürnberg (FAU), Department of Dermatology, STIs, and Allergy, University Hospital Erlangen, Erlangen, Germany; 2 Division of Infectious Diseases, Department of Medicine, Sunnybrook Health Sciences Centre, University of Toronto, Toronto, Canada; 3 Centers for Disease Control and Prevention, Kisumu, Kenya; 4 IPA, Innovations for Poverty Action, Busia, Kenya; University of Cincinnati School of Medicine, UNITED STATES

## Abstract

**Background:**

Measuring effectiveness of HIV prevention interventions is challenged by bias when using self-reported knowledge, attitude or behavior change. HIV incidence is an objective marker to measure effectiveness of HIV prevention interventions, however, because new infection rates are relatively low, prevention studies require large sample sizes. Herpes simplex virus type 2 (HSV-2) is similarly transmitted and more prevalent and could thus serve as a proxy marker for sexual risk behavior and therefore HIV infection.

**Methods:**

HSV-2 antibodies were assessed in a sub-study of 70,000 students participating in an education intervention in Western Province, Kenya. Feasibility of testing for HSV-2 antibodies was assessed comparing two methods using Fisher’s exact test. Three hundred and ninety four students (aged 18 to 22 years) were randomly chosen from the cohort and tested for HIV, *Chlamydia trachomatis*, *Neisseria gonorrhoeae*, and *Trichomonas vaginalis*. Out of these, 139 students were tested for HSV-2 with ELISA and surveyed for sexual risk behavior and 89 students were additionally tested for HSV-2 with a point-of-contact (POC) test.

**Results:**

Prevalence rates were 0.5%, 1.8%, 0.3% and 2.3% for HIV, *Chlamydia trachomatis*, *Neisseria gonorrhoeae*, and *Trichomonas vaginalis*, respectively. Prevalence of HSV-2 antibodies was 3.4 % as measured by POC test (n=89) and 14.4 % by ELISA (n=139). Specificity of the POC test compared with ELISA was 100%, and the sensitivity only 23.1%. Associations between self-reported sexual behavior and HSV-2 serostatus could not be shown.

**Conclusions:**

Associations between self-reported sexual risk behavior and HSV-2 serostatus could not be shown, probably due to social bias in interviews since its transmission is clearly linked. HSV-2 antibody testing is feasible in resource-poor settings and shows higher prevalence rates than other sexually transmitted diseases thus representing a potential biomarker for evaluation of HIV prevention interventions.

## Introduction

Herpes simplex virus type 2 (HSV-2) is transmitted almost exclusively sexually. After initial infection the virus persists in the sensory ganglia for life. This latent infection can be reactivated to induce recurrent disease. HSV-2 is one of the most prevalent sexually transmitted infections (STI) [1,2]. After initial infection individuals develop antibodies that are detectable throughout life. The seroprevalence of HSV-2 antibodies varies greatly by gender, age group, country, region within the country, and across subpopulations [3]. Once adolescents become sexually active, a sharp increase in prevalence of HSV-2 antibodies has been observed in various studies [3,4] rising from, for example, 5% in a population-based sample of males 13–14 years of age, to 60% in males 25–29 years of age, in a study conducted in western Kenya; women had higher rates of 10% and 90%, in the respective age groups [5].

HSV-2 is also increasingly important as cause for genital ulcer disease (GUD) [6] and as a cofactor in HIV infection [5,7,8]. Conversely, HIV acquisition is associated with HSV-2 seropositivity [7], and GUD is increased after HIV seroconversion. If genital ulcerations are present, the HIV transmission rate is 7 to 11 times higher [9,10]. Worldwide about 90 to 100% of HIV positive individuals are co-infected with HSV-1 and about 52 to 95% with HSV-2 [11]. Not only symptomatic HSV-2 infections with ulcerations but also latent infections without any clinical signs lead to a higher HIV susceptibility through activation of immunological mechanisms [12].

HIV seroprevalence as well as HSV-2 seroprevalence has been shown to be closely associated with sexual behavior [13,14]; but prevalence for HSV-2 is much higher than for HIV [15]. HIV incidence is the best outcome measure of HIV prevention programs. However, in the evaluation of HIV intervention in low prevalence settings, its use may be limited by low incidence rates that would require large sample sizes to show differences between intervention groups. Subjective and indirect markers of self-reported sexual behavior are often used as a proxy marker in order to determine an HIV intervention’s success. However, these markers are prone to error due to role expectations, shame and/or social desirability bias [16] and thus can be misleading. It is known that survey data does not adequately reflect sexual behavior, especially not data gathered in structured interviews [17]. To determine effectiveness of HIV prevention interventions, measurement of objective markers is crucial.

After infection with HSV-2, seroconversion occurs and can be detected by an ELISA (enzyme linked immunosorbent assay) test from serum. HSV type-specific ELISAs based on recombinant glycoprotein G1 (gG1, antigen) and recombinant glycoprotein G2 (gG2, antigen) have been approved by the US Food and Drug Administration (FDA) for use in adult populations and have been shown to have a high sensitivity and specificity in developed countries (97% and 95%, respectively) [18,19]. However, ELISA as well as Western Blot are sophisticated testing methods that can only be reliably perfomed in laboratories with a high technical standard. Moreover, they are costly and time-consuming. In the last few years an alternative rapid point-of-contact (POC) testing method has become available.

The aim of this study was (i) to evaluate the feasibility of HSV-2 antibodies as a biomarker for cumulative sexual risk behavior in adolescents and (ii) to assess the prevalence of HSV-2 antibodies compared to other STIs. Furthermore, to assess methods of HSV-2 testing, the study compared the POC test to the gold standard ELISA, in a low-resource setting.

## Methods

### Study Setting and Population

Between 2003 and 2006, researchers from Innovation for Poverty Action, Kenya (IPAK), ran a large-scale randomized HIV prevention intervention in primary schools in the Western Province of Kenya with the non-profit organization International Child Support (ICS) [20]. The project included 4 components: 1) informing teenagers of the heightened risk associated with cross-generational sex; 2) promoting debates and essay writing on methods of protection against HIV infection; 3) reducing the cost of education and 4) the national teacher training program. Altogether 328 schools with all students in grades 5 to 8 participated in the project. All of them received the national AIDS education program, and some were randomly selected to participate in one or more of these four additional interventions [21]. Upon follow-up for this HSV-2 study students had aged to 18 to 22 years.

### Data Collection

A total of 785 students (413 girls, 372 boys) were randomly sampled from the 20,000 students enrolled in grades 7 and 8 in 2003 [21]. In total 64% of sampled students were found, contacted in their homes and asked to participate in this prospective study. After giving informed consent, 394 students were tested for HIV, *Chlamydia trachomatis*, *Neisseria gonorrheae* and *Trichomonas vaginalis*. A subset of students were informed about the substudy for HSV-2 testing which took place in the health center closest to the student’s homes in Bumula, Mechemeru, Miendo, and Bungoma, all in Western Province, Kenya. On the days the testing was done in total 139 students presented to the health centers and were given detailed information on the procedure. After giving informed consent they were surveyed by field workers for health-seeking and sexual behavior and blood was drawn for the ELISA by nurses under the supervision of a medical doctor (LH). The POC test was performed on the first 89 students, using capillary blood from a finger which was performed according to manufacturer’s instructions by VCT (voluntary counseling and testing) counselors.

If symptoms of STIs were present at the time of the visits, free syndromic treatment was employed according to the Kenyan STI treatment guidelines with free medical treatment and counseling. All STI results obtained during the study, including HSV-2, were reported to respondents. Those with a positive test result received organism specific treatment, also free of charge, as per the Kenyan STI treatment guidelines. If follow-up was needed, respondents were encouraged to seek medical attention or further testing at the local STI clinic or healthcare facility free of charge. The STI clinics were also provided with the drugs for treatment and participants were provided with vouchers to receive free treatment at the nearest STI clinic.

### Confidentiality

Numeric identifiers were used on all documents containing respondent’s information. Respondents were provided with their ID number to find out about their test results and receive STI treatment in case of symptoms or positive results.

### Survey

The survey was conducted as a structured interview in Swahili and contained the following sections:

- Symptoms and follow-up on referral
- Follow-up on test results
- Follow-up on a previous condom intervention
- Behavior changes as general consequences to counseling
- Treatment

The focus of analyses was behavior change related to risky sexual behavior. We studied characteristics of sexual behavior like truthfulness, number of sex partners, unprotected intercourse, risk awareness, and information to the partner in case of STI as well as health seeking behavior (S1 Table). All participants answered the given interview questions in a private surrounding.

### HSV-2 testing

To assess HSV-2 antibodies two methods were employed: (1) The POC rapid testing using capillary blood obtained by finger prick and (2) the analysis by ELISA in serum samples. For the former the FDA-approved (2007) POC test kit HerpeSelect Express (Focusdiagnostics, Cypress, CA, US) was used. After disinfection, the finger tip was punctured with a sterile lancet. Blood was collected using the supplied capillary tubes and applied to the test kits using the plunger also provided in the kits. After waiting 30 seconds, the kits were opened and buffer applied according to manufacturer’s instructions. Kits were read after 15 minutes and results recorded. All these procedures were done at the VCT site.

For analysis by ELISA, venous blood was drawn and serum separated and cryopreserved at the collection site. Serum probes were analyzed for anti-IgG- antibodies using the HerpeSelect-2-ELISA (Focusdiagnostics, Cypress, CA, US). Samples were tested at AMPATH Laboratory at Moi University in Eldoret Kenya. ELISA was carried out according to manufacturer’s instructions as described by Ashley-Morrow *et al*. [4]. HerpeSelect-2-ELISA was considered positive if the index value exceeded 3.5. For the results with index values from 1.1–3.5 testing was repeated. Index values that remained between 1.1–3.5 were excluded from the analysis.

### HIV testing

All three components of voluntary counseling and testing (VCT) for HIV (pre-test counseling, HIV testing and post-test counseling) were performed in a single visit, as practiced in national HIV VCT sites in Kenya. Field workers were trained by the Kenya Association of Professional Counselors and certified by NASCOP to deliver full VCT services. The rapid test DetermineHIV-1/2 (Alere GmbH, Cologne, Germany) was used. Testing was performed by finger prick with a sterile lancet to obtain two drops of blood and the result could be read after 15 minutes. Additionally, all participants received another rapid test (BiolineHIV-1/2, Standard Diagnostic Inc., Kyonggi-do, South Korea). Participants with discordant first and second test results underwent a third rapid, tiebreaker test (Uni-Gold Recombigen HIV, Trinity Biotech PLC, Wicklow, Ireland). Dry blood spots were collected for participants who provided consent for testing but declined to know their HIV results and on every 10^th^ respondent for quality assurance. Dry blood spots were tested at the Medical Research Institute/Center for Disease Control (KEMRI/CDC) in Kisumu, Kenya.

### Detection of *Trichomonas vaginalis*

InPouchTV testing kits (Biomed Diagnostics Inc, White City, Oregon, US) were used for testing of *Trichomonas vaginalis* (TV) on vaginal culture samples for women and on urine samples for men. Vaginal culture samples were collected through self-administered swabs. Urine samples were collected after respondents had held the urine for at least one hour. First-catch urine was collected in sterile screw-capped urine collection containers. Urine samples were centrifuged and a probe taken with a sterile swab to inoculate the InPouchTV testing kit (Biomed Diagnostics, San Jose, CA). Inoculated test pouches were transported to the nearest laboratory where they were observed in wet-mount technique. They were incubated at 37°C for three days. Examination for TV test result was carried out every 24 hours according to the manufacturer’s protocol.

### Testing for *Neisseria gonorrhoeae*

The InTray GC testing kits (Biomed Diagnostics Inc, White City, Oregon, US) were used for detection of *Neisseria gonorrhoeae* vaginal samples for women. Culture plates were inoculated using vaginal swabs and incubated for 24 up to 48 hours at 37°C. In addition, Polymerase Chain Reaction (PCR) tests were performed on urine samples of men and women. Urine samples were collected after respondents had held the urine for at least one hour. First-catch urine was collected in sterile screw-capped urine collection containers. The urine samples were kept at 4°C and transported to the laboratory where the samples were stored at -20°C. Examination was carried out after 24 hours according to manufacturer’s protocol.

### Testing for *Chlamydia trachomatis*

PCR tests were performed for *Chlamydia trachomatis* on urine samples of men and women. All PCR tests were performed at AMPATH Lab at Moi University in Eldoret, Kenya, as described previously [22].

### Data analyses

All data were anonymized. All test results and survey data were double-entered and validated. Data was analyzed using Stata version 9.0 and SPSS 17.0. Associations between HSV-2 serostatus and self-reported sexual behavior were analyzed using categorical variables resulting from HerpeSelect-2-ELISA and survey data. Cross tabulations were created and Fisher’s exact test was done to assess if anticipated correlation were existing. Descriptive and unadjusted data analysis has been done. Therefore p-values are descriptive; a p-value < 0.05 indicates an association between HSV-2 serostatus and sexual behavior. Analyses were done including both genders and gender-specific.

Comparison of HerpeSelect Express to HerpeSelect-2-ELISA was done calculating the sensitivity and specificity, including 95% CI, as well as the concordance. HerpeSelect-2-ELISA was chosen as the reference test since it has been used extensively and compared to other HSV-2 tests in different settings worldwide.

### Ethics

Approval of the study was granted in a written form by the Kenya National Ethical Review Committee located at the Kenya Medical Research Institute (KEMRI), by the Ministry of Health, by the Ministry of Education, as well as the institutional review boards (IRBs) of the institutions involved in the study (Innovations for Poverty Action-Kenya Institutional Review Board, Massachusetts Institute of Technology (MIT) Committee on the Protection of Human Subjects). Local authorities also approved the trial (Provincial and District health officials). Participants provided their written informed consent to participate in this study.

## Results

In total, 394 individuals were tested for HIV, *Neisseria gonorrhoeae*, *Chlamydia trachomatis*, and *Trichomonas vaginalis*. A randomly chosen subsample of 139 students was tested for HSV-2 with ELISA and surveyed for health-seeking and sexual behavior. Out of these, 89 individuals additionally underwent HSV-2 testing with the POC test.

### Prevalence of STIs

HIV prevalence in our sample was 0.5% (2 out of 394; 95% CI 0% to 1.2%). HSV-2 antibodies measured by ELISA showed a prevalence of 14.4% (20 out of 139). Prevalence for *Trichomonas vaginalis*, *Chlamydia trachomatis* and *Neisseria gonorrhoeae* was 2.3% (9 out of 394), 1.8% (7 out of 394) and 0.3% (1 out of 394), respectively (see Table 1).

**Table 1:** Prevalences of sexually transmitted diseases.

	male positive (%)	female positive (%)	total positive (%)
HIV(n = 394)	0.0	1.0	0.5
*Chlamydia trachomatis*(n = 394)	2.0	1.5	1.8
*Neisseria gonorrhoeae*(n = 394)	0.0	0.5	0.3
*Trichomonas vaginalis*(n = 394)	1.0	3.6	2.3

### HSV-2 serology: POC compared to ELISA

Testing for HSV-2 antibodies showed 3.4% positive test results by HerpeSelect Express (POC) and 14.4% by HerpeSelect-2-ELISA. POC test sensitivity in comparison with the gold standard ELISA was 23.1% (95% CI 15.4% to 32.9%); specificity was 100.0% (95% CI 95.3% to 100.0%). Concordance of the POC-kit with ELISA was 88.8% (see Table 2).

**Table 2:** HerpeSelect Express POC test compared to HerpeSelect-2-ELISA. Correlation of positive and negative findings, n = 89.

		ELISA	
		negative n (%)	positive n (%)
**POC**	negative	76 (100.0)	10 (76.9)
	positive	0 (0.0)	3 (23.1)
Sensitivity (95% CI): 23.1% (15.4%- 32.9%)			
Specificity (95% CI): 100% (95.3%- 100%)			
Concordance rate: 88.8%			

### Health-seeking and behavioral data

In order to assess associations between self-reported data on behavior and HSV-2 serostatus, questions on health-seeking and sexual behavior were analyzed. Fisher’s exact test showed no associations between HSV-2 serostatus and sexual behavior stated in interviews (S1 Table). In our sample the two students who had a positive HIV status also showed HSV-2 seropositivity.

## Discussion

Serological testing for HSV-2 is feasible in resource-poor settings, and testing of HSV-2 antibodies resulted in a positive rate of HSV-2 antibodies of 14.4% as measured by ELISA in serum probes. This rate was much higher than the prevalence rate for HIV, which was 0.5%, or for *Trichomonas vaginalis*, *Chlamydia trachomatis* and *Neisseria gonorrhoeae*, which was 2.3%, 1.8% and 0.3%, respectively.

Of note, HIV and HSV-2 prevalence data as well as other STI rates were much lower than data from prior STI prevalence studies conducted in the same age group in rural Western Kenya [5] where HSV-2 prevalence was around 40% and HIV prevalence around 15% in a study group of adolescents aged 18 to 22 [5]. In another study conducted in women aged 18–50 in six rural communities in Kenya, prevalence rates for STIs were 1.3–6.1% for gonorrhoea, 1.2–5.0% for chlamydia and 10.6–29.9% for trichomoniasis. Overall STI prevalence ranged from 13.4% to 31.2% [23]. In a health center-based study in Western Kenya the prevalence rates observed were 3.8%, 6.3%, and 10.9% for gonorrhea, chlamydia and trichomoniasis, respectively [24]. The lower STI infection rates in our study may be due to improvement in STI services and the concurrent impact of large-scale HIV service and anti-retroviral therapy roll out in sub-Saharan Africa over the last decade [25]. This favorable development, limits the ability to evaluate HIV interventions using HIV incidence as the primary outcome, because in these low HIV prevalence settings, large studies would be needed to provide enough statistical power to detect impacts of a specific intervention even in a randomized design. HSV-2 shows higher prevalence rates than HIV in our study and other studies [5]. Furthermore, HSV-2 infection is correlated with HIV infection. In areas with low HSV-2 prevalence, HIV rates are highest in high risk groups, whereas in areas with high HSV-2 prevalence generalized HIV transmission can be seen [26,27]. Therefore, HSV-2 has been discussed as a proxy biomarker in order to evaluate effectiveness of HIV intervention studies [28].

### HSV-2 POC Antibody Testing in comparison to ELISA

HerpeSelect-2-ELISA has previously shown a high sensitivity and specificity of 97% and 95% [18] and HerpeSelect Express-POC a sensitivity of 96% and a specificity of 98% [29], both in adult populations in developed countries. HerpeSelect Express-POC compared to HerpeSelect-2-ELISA achieved a sensitivity of 100% and a specificity of 98% when the cutoff value for the ELISA was set to 3.5 in an adult population in the USA [30]. Within our study setting in rural Kenya, specificity of the POC-test was also high with 100%. However, sensitivity was only 23%, which highlights a need as suggested by others [31] for further evaluation of HSV-2 POC testing in African populations.

There are clear advantages for the POC test in a decentralized low-resource setting as HSV-2 testing could then be done in both the facility and the household level. There is no need for electricity, testing expenses are lower compared to ELISA, and counselors can easily be trained. However, the low sensitivity compared with data from developed countries remains a concern to be addressed. There are three potential reasons for the low sensitivity in our study.

Firstly, ELISA seems to be more sensitive in early seroconversion than POC (and Western Blot) [4,32,33] which might play a role in this study population that was rather young with a frequent occurrence of seroconversion. Furthermore, several studies have demonstrated that HerpeSelect-2-ELISA’s sensitivity and especially specificity in South and East African countries was lower than in industrial countries and that there were differences between African countries themselves [4,32]. Some authors propose that HerpeSelect-2-ELISA is more sensitive than Western Blot in an early infection phase with low antibody titers and therefore detects early seroconversion more accurately than other testing methods [33,34]. Since HSV-2 prevalence is much higher in African countries than in industrial countries, where ELISA has been evaluated and approved, it is possible that discordant results in testing methods are based on a higher sensitivity in an early stage of infection. Therefore, HerpeSelect-2-ELISA in contrast to Western Blot should not be seen as less accurate but because of its high sensitivity as possibly more reliable. This theory is supported by one study demonstrating many false positive results in ELISA compared to Western Blot exclusively within the African sample; HSV-2 prevalence rate was 62% and therefore seroconversion is a frequently occurring event [4].

Secondly, antibody cross-reactivities within HerpeSelect-2-ELISA might exist to other antibodies and thus lead to false positive results in ELISA. Antibodies against HSV-1, Varicella-Zoster virus or HIV could lead to an increased number of false positive results with HerpeSelect-2-ELISA when testing for HSV-2 [34]. One study noticed a decrease in ELISA’s specificity from 61% to 30% in HIV-1 co-infected individuals, meaning that ELISA showed more false positive and therefore less true negative results [35]. However, other studies found no associations between decrease of specificity and HIV status [36,37]. If existing, such cross-reactions could partly explain our high rate of positive results in ELISA. HerpeSelect Express POC test should then not be seen as less accurate than ELISA.

Finally, geographical strain variation of HSV-2 could influence testing results. HerpeSelect-2-ELISAs have been evaluated in industrial countries (mostly USA) and have shown a high sensitivity of 97% and specificity of 95% in adult populations compared to the well-established Western Blot [18,19]. Therefore, HerpeSelect-2-ELISA was chosen as the comparator in this study. Rapid POC assays have also been evaluated in developed countries and showed a similarly high sensitivity and specificity, so that they are said to be comparable to the well-established methods ELISA and Western Blot [29,30]. Geographical differences in the reliability of testing methods have been noticed when comparing them in various populations [4,37,38,39]. Sensitivity and specificity of ELISA compared to Western Blot were shown to be lower in South and East Africa than in other nations [35], i.e. 93% specificity compared to Western Blot in nine nations, but only 70% in Nigeria [4]. This means ELISA showed more positive results in Nigeria than Western Blot did.

In this Kenyan study group, ELISA possibly detects a bigger diversity of HSV-2 strain specific antibodies than the POC test does. This could explain the POC test’s low sensitivity compared to ELISA in this study. Worldwide analyses of HSV-2 strain variability are necessary in order to evaluate the theory that HSV-2 strain variations could be the reason for discordant test results in different countries.

### HIV and HSV-2 in association to risky sexual behavior and attitudes

Self-reported sexual behavior is often used as an indirect proxy marker to evaluate HIV infection risk. However, subjective reporting is limited by social desirability bias [16]. Indeed, we found no association between HSV-2 serostatus and self-reported sexual behavior, probably due to inaccuracy in subjective self-report, as described before [17]. All subjects in our sub-study had previously participated in an HIV prevention program, so they might have been tempted to give study investigators socially expected answers, as opposed to responses that accurately reflected actual behavior. In this study error-prone self-reported sexual behavior leads to limited conclusions in the analysis of correlations between HSV-2 serostatus and sexual risk behavior. Subjective markers are limited in evaluating HIV interventions, and our study further supports the use of objective biomarkers like HSV-2 antibodies [31]. Indeed, other studies looking at prevention interventions (e.g. conditional cash transfer) [40] also report associations between sexual behavior changes, and HIV as well as HSV-2 serostatus [40]. In our study, the two students with positive HIV status also showed HSV-2 seropositivity. The sample size and HIV prevalence were both too low to allow to draw conclusions or to perform analysis with enough statistical power. Huge studies with large sample sizes would be needed for such an evaluation.

## Conclusions

Anticipated associations between positive HSV-2 serostatus (proxy marker) and sexual risk behavior could not be shown, probably due to social bias in interviews since its transmission is clearly linked. Therefore, HSV-2 should be considered as a potential biomarker for cumulative sexual risk behavior, however large studies regarding direct associations between HSV-2 and HIV serostatus are needed. The assessment of HSV-2 antibodies was subsequently employed for the large HIV intervention evaluation [21]. HSV-2 antibody testing can be performed in resource-poor settings and HSV-2 antibodies have a higher prevalence than other sexually transmitted diseases and thus represent a feasible biomarker for evaluation of HIV intervention programs. Further investigations are needed regarding optimal HSV-2 testing methods in decentralized low-resource settings in the region.

## Supporting Information

S1 Table Analysis of health-seeking and sexual behavior in association to HSV-2 serostatus in 139 subjects. (DOC)
