# Supplementary material for: Evaluating HIV Prevention Programs: Herpes Simplex Virus Type 2 Antibodies as Biomarker for Sexual Risk Behavior in Young Adults in Resource-Poor Countries
Source: PLoS One. 2015 May 26;10(5):e0128370. doi: 10.1371/journal.pone.0128370 (PMC4444314; doi:10.1371/journal.pone.0128370)
Supplement: S1 Table — (DOC) [file pone.0128370.s001.doc]

**S1 Table: Analysis of health-seeking and sexual behavior in association to HSV-2 serostatus in 139 subjects.**

|  | **HSV-2 serostatus** | | | | |
| --- | --- | --- | --- | --- | --- |
|  | positive, n (% ) | | negative, n (%) | | p-value |
| **Unfaithfulness (Question 54.1)** |  |  |  |  |  |
| Collectively |  |  |  |  | 1.0 |
| *Yes* | 4 | (20.0) | 22 | (18.5) |  |
| *No* | 16 | (80.0) | 97 | (81.5) |  |
| Male |  |  |  |  | 0.432 |
| *Yes* | 4 | (63.4) | 10 | (20.8) |  |
| *No* | 7 | (63.6) | 38 | (79.2) |  |
| Female |  |  |  |  | 0.342 |
| *Yes* | 0 | (0.0) | 12 | (16.9) |  |
| *No* | 9 | (100.0) | 59 | (83.1) |  |
| **≥ 1 sexual partner (Question 54.2)** |  |  |  |  |  |
| Collectively |  |  |  |  | 0.196 |
| *Yes* | 3 | (15.0) | 8 | (6.7) |  |
| *No* | 17 | (85.0) | 111 | (93.3) |  |
| Male |  |  |  |  | 1.0 |
| *Yes* | 1 | (9.1) | 5 | (10.4) |  |
| *No* | 10 | (90.9) | 43 | (89.6) |  |
| Female |  |  |  |  | 0.095 |
| *Yes* | 2 | (22.2) | 3 | (4.2) |  |
| *No* | 7 | (77.8) | 68 | (95.8) |  |
| **Unprotected intercourse (Question 54.3)** |  |  |  |  |  |
| Collectively |  |  |  |  | 0.692 |
| *Yes* | 1 | (5.0) | 13 | (10.9) |  |
| *No* | 19 | (95.0) | 106 | (89.1) |  |
| Male |  |  |  |  | 0.67 |
| *Yes* | 1 | (9.1) | 9 | (18.8) |  |
| *No* | 10 | (90.9) | 39 | (81.3) |  |
| Female |  |  |  |  | 1.0 |
| *Yes* | 0 | (0.0) | 4 | (5.6) |  |
| *No* | 9 | (100.0) | 67 | (94.4) |  |
| **Low awareness of risk (Question 55.1)** |  |  |  |  |  |
| Collectively |  |  |  |  | 0.468 |
| *Yes* | 10 | (50.0) | 48 | (40.3) |  |
| *No* | 10 | (50.0) | 71 | (59.7) |  |
| Male |  |  |  |  | 0.2 |
| *Yes* | 8 | (72.7) | 24 | (50.0) |  |
| *No* | 3 | (27.3) | 24 | (50.0) |  |
| Female |  |  |  |  | 0.71 |
| *Yes* | 2 | (22.2) | 24 | (33.8) |  |
| *No* | 7 | (77.8) | 47 | (66.2) |  |
| **High awareness of risk (Question 56.1)** |  |  |  |  |  |
| Collectively |  |  |  |  | 0.288 |
| *Yes* | 5 | (25.0) | 41 | (34.5) |  |
| *No* | 15 | (75.0) | 78 | (65.5) |  |
| Male |  |  |  |  | 1.0 |
| *Yes* | 2 | (18.2) | 10 | (29.8) |  |
| *No* | 9 | (91.8) | 38 | (79.2) |  |
| Female |  |  |  |  | 0.726 |
| *Yes* | 3 | (33.3) | 31 | (43.7) |  |
| *No* | 6 | (66.7) | 40 | (56.3) |  |
| **Informing partner in order to prevent transmission (Question 27.4)** |  |  |  |  |  |
| Collectively |  |  |  |  | 1.0 |
| *Yes* | 0 | (0.0) | 3 | (2.5) |  |
| *No* | 20 | (100.0) | 116 | (97.5) |  |
| Male |  |  |  |  | 1.0 |
| *Yes* | 0 | (0.0) | 1 | (2.1) |  |
| *No* | 11 | (100.0) | 47 | (97.9) |  |
| Female |  |  |  |  | 1.0 |
| *Yes* | 0 | (0.0) | 2 | (2.8) |  |
| *No* | 9 | (100.0) | 69 | (97.2) |  |
| **Openness towards the partner about STI results (Question 27.6)** |  |  |  |  |  |
| Collectively |  |  |  |  | 0.546 |
| *Yes* | 1 | (5.0) | 4 | (3.4) |  |
| *No* | 19 | (95.0) | 115 | (96.6) |  |
| Male |  |  |  |  | 0.468 |
| *Yes* | 1 | (9.1) | 2 | (4.2) |  |
| *No* | 10 | (90.9) | 46 | (95.8) |  |
| Female |  |  |  |  | 1.0 |
| *Yes* | 0 | (0.0) | 2 | (2.8) |  |
| *No* | 9 | (100.0) | 69 | (97.2) |  |
| **Visiting traditional healer (Question 18)** |  |  |  |  |  |
| Collectively |  |  |  |  | 1.0 |
| *Yes* | 0 | (0.0) | 2 | (18.2) |  |
| *No* | 2 | (100.0) | 9 | (81.8) |  |
| Male |  |  |  |  | 1.0 |
| *Yes* | 0 | (0.0) | 1 | (2.1) |  |
| *No* | 11 | (100.0) | 47 | (97.9) |  |
| Female |  |  |  |  | 1.0 |
| *Yes* | 0 | (0.0) | 1 | (1.4) |  |
| *No* | 9 | (100.0) | 70 | (98.6) |  |
| **Taking traditional medicine (Question 19)** |  |  |  |  |  |
| Collectively |  |  |  |  | 1.0 |
| *Yes* | 0 | (0.0) | 2 | (1.7) |  |
| *No* | 20 | (100.0) | 117 | (98.3) |  |
| Male |  |  |  |  |  |
| *Yes* | 0 | (0.0) | 0 | (0.0) | 1.0 |
| *No* | 11 | (100.0) | 48 | (100.0) |  |
| Female |  |  |  |  | 1.0 |
| *Yes* | 0 | (0.0) | 2 | (2.8) |  |
| *No* | 9 | (100.0) | 69 | (97.2) |  |
